# Locomotor Inhibition in Adult Horses Faced to Stressors: A Single Postpartum Experience May be Enough!

**DOI:** 10.3389/fpsyg.2012.00442

**Published:** 2012-10-29

**Authors:** Virginie Durier, Séverine Henry, Carol Sankey, Jacques Sizun, Martine Hausberger

**Affiliations:** ^1^UMR 6552 “Ethologie Animale et Humaine,” CNRS-Université de Rennes 1Rennes, France; ^2^Pôle de la Femme, de la Mère et de l’Enfant, Centre Hospitalo-UniversitaireBrest, France

**Keywords:** behavior, early experience, neonatal handling, stress reaction, locomotor activity

## Abstract

Despite the number of postpartum handling that a newborn experiences, few studies focus on their long-term consequences. In rats, regular long separations from the mother, during the early life, led to modifications of the locomotor activity when the animal is confronted to a stressor. In horses, one component of the behavioral response to stressful situation is active locomotion. We wondered if the routine postpartum handling undergone by foals, would affect their level of reactivity or the way they express their stress, when older. One single prolonged bout of handling just after birth clearly affected later adult expression of stress reactivity. In social separation associated with novelty, handled, and unhandled horses produced an equal amount of whinnies, showing a similar vocal response to stress. However, both groups differed in their locomotor response to the situations. Early handled foals expressed less of the active forms of locomotion than the control group. Our findings highlight the need of further reflections on long-term effects of routine handlings procedures close to birth.

## Introduction

Although highly suspected, long-term effects of postpartum experience on behavior and emotions are rarely demonstrated for different reasons. One is the duration of long-term developmental studies; another is the interference of uncontrolled factors during development and their potential resilience effects. Thus, while the impact of early care of babies and their associated experience (being separated from their mother, handled, having suckled, or not during the first hour after birth) is questioned (Klaus and Kennell, 2001) with observable short-term effects (Long et al., 1980; Jansson et al., 1995; Bystrova et al., 2003), some scarce studies on potential long-term effects indicate impacts of postpartum experience still visible in 3- or 5-year-old children (Ringler et al., 1978; Wiberg et al., 1989).

Most evidence of long-term effects of early experience comes from laboratory rats and monkeys and involves maternal separation (see review in Loman and Gunnar, 2010). Even short and temporary separations induce durable effects on emotional reactivity and social competencies (Rhesus monkeys: Spencer-Booth and Hinde, 1971; rats: Weinberg et al., 1978). The famous monkey models of deprived animals are well represented by hunched postures and reduced mobility (Harlow et al., 1965; Kraemer, 1992). Increased immobility when facing a stressor (forced swim test) was also observed in rats that experienced regular long separations from their mothers during their two first weeks of life (Lehmann and Feldon, 2000).

Immobility or locomotor inhibition are observed regularly in stressful situations and are traits included in concepts such as “conservation-withdrawal” or “learned helplessness” (Engel and Schmale, 1972; Maier and Seligman, 1976). Both correspond to situations when inputs become excessive and beyond the organism’s capacity to cope actively with the stressors, e.g., removal of mother (Kaufman and Rosenblum, 1967). In the second case, the animal has learned that trying actively to escape the situation is useless. Separation from the attachment object leads first to protest (active seeking of contact) and then to despair, characterized by activity decline (Bowlby, 1969; Hofer, 1994).

Postpartum handling may require separation from the mother at a very early stage, and intensive handling (bathing, weighting, eye ointment…) that persists even if the infant protests by crying or agitating its arms and legs (Bystrova et al., 2003). This is a typical situation when the individual has no control over the situation and this questions the possibility of its effects on later reaction modalities to stressful situations.

To test this hypothesis, “naturalistic” animal models with individuals that experience impaired maternal care and social living are required. Previous studies show that horses, a species with a strong dam-foal bond, are highly sensitive to postpartum experience. Thus, “routine” handling of foals at birth has short-, medium-, and long-term effects on their attachment to their dam and their social development. Indeed, horses that have been handled at birth spend longer time close to their mothers and interact less often with peers than unhandled individuals, from early stages of development to “adolescence” (Henry et al., 2009). Even a slight interference during first suckling has similar consequences on attachment (Hausberger et al., 2007). The way they are handled at birth influences their emotional reactivity at later stages (de Boyer des Roches et al., 2011). When handling was performed only on one side of the body, the reactions of 10-day-old foals to a human approach differed according to the side stimulated. Foals handled on the right were fewer to accept contact with humans (long delay, contact avoidance). Long-term effects of a single early experience on adult behaviour in this species could be demonstrated despite their “normal” ecological living conditions in the meanwhile.

In the present study, we hypothesized that postpartum handling, which consists in maintaining the foal immobile and rubbing its body all over, would induce a form of learned helplessness (Simpson, 2002) that would have consequences on its later ability, as an adult, to react actively to stressful situations. During this neonatal handling, foals react first by struggling (“protest”) and then remain motionless (“despair”; Simpson, 2002; Henry et al., 2009). They may thus have learned that motor behavior is useless to escape a stressful situation (inescapable stress). Therefore, we compared the reactions of early handled foals and of control (unhandled) foals to stressful situations when one (early adolescence) and 2 (pre-adult stage) years old. Previous studies showed that horses’ reactions to stress include postural, vocal, and locomotor components (Wolff et al., 1997; Visser et al., 2001). Following social separation, horses tend to seek restoration of contact actively through whinnies and active locomotion (trot, canter). Whinnies are commonly used as a marker of stress, above all in social separation situations (Harewood and McGowan, 2005; Pond et al., 2010; Yeon, 2012). We expected the locomotor pattern to be affected by their early experience of inescapable stress while the vocal component should not be affected.

## Results

At birth, foals are routinely handled by a human following a standard procedure consisting in maintaining the foals in a recumbent position on the floor and stroking it all over its body for about 1 h. Early handled and unhandled (control) foals were observed in stressful situations: social isolation in familiar and unfamiliar settings and presence of a novel object, when they were one and 2 years old.

**Figure 1:**
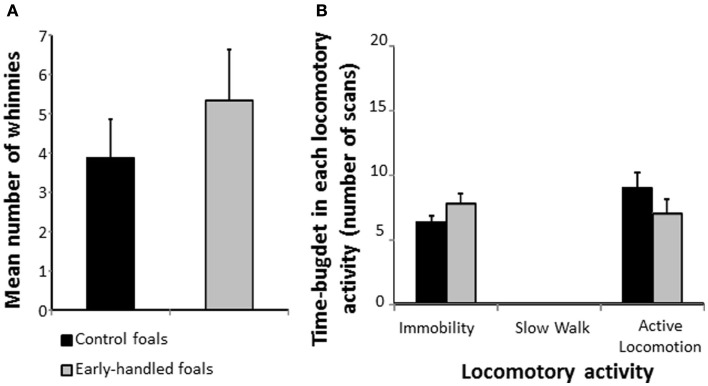
**Behaviors of 1-year-old, early handled, and unhandled foals during social separation**. Foals were left alone in their familiar stall. **(A)** Mean number (±SEM) of whinnies emitted during the experiments; **(B)** mean number (±SEM) of scans when the foals performed different types of locomotor activity.

**Figure 2:**
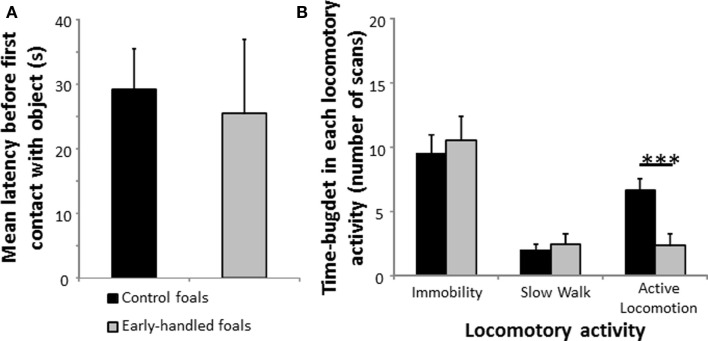
**Reaction to novelty of 1-year-old, early handled, and unhandled foals**. **(A)** Mean latency (±SEM) before the first contact with the novel object. **(B)** Mean number (±SEM) of scans when the foals performed different forms of locomotor activity.

One-year-old early handled and control foals reacted strongly to social isolation in their familiar stall by whinnying. There was no difference in the number of whinnies between the two groups (Mann–Whitney U test: *N*_early handled_ = 9; *N*_control_ = 8; *z* = 0.73, *p* = 0.48; Figure 1A). Level of locomotor activity did not differ significantly between the two groups (Mann–Whitney U tests: *N*_early handled_ = 9; *N*_control_ = 8; immobility: *z* = 1.08, *p* = 0.28; active locomotion: *z* = −1.17, *p* = 0.29; Figure 1B). When foals were confronted to a novel object, latencies to approach the object did not differ significantly between groups (Mann–Whitney U test: *N*_early handled_ = 9; *N*_control_ = 8; *z* = −1.06; *p* = 0.28; Figure 2A). Nevertheless, the control group’s level of locomotor activity was significantly higher when a novel object was presented (Mann–Whitney U test: *N*_early handled_ = 9; *N*_control_ = 8; immobility: *z* = 0.48, *p* = 0.6; slow walk: *z* = −0.1, *p* = 0.89; active walk: *z* = −2.66, *p* = 0.005; Figure 2B).

**Figure 3:**
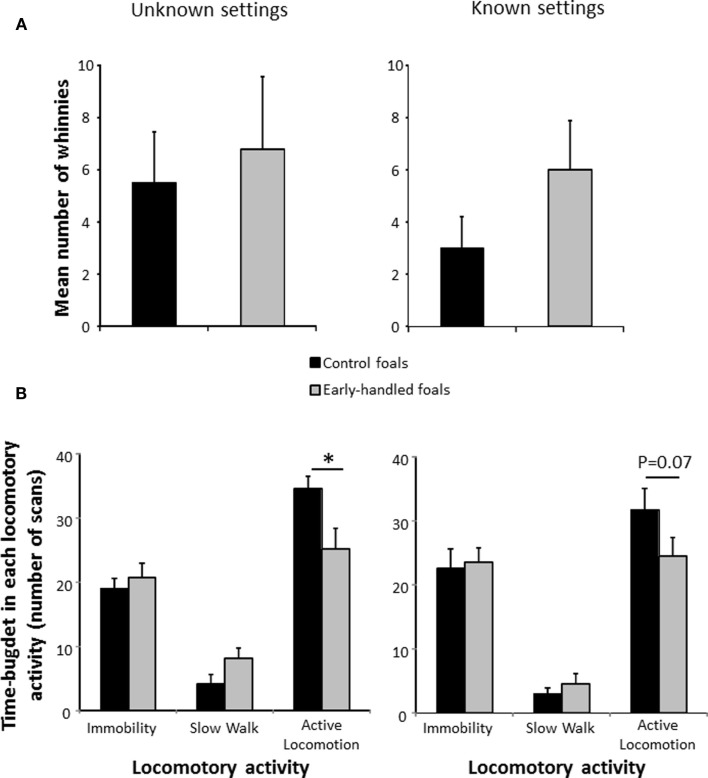
**Behaviors of 2-year-old, early handled, and unhandled foals during social separation**. **(A)**: mean number (±SEM) of whinnies emitted during the experiments; **(B)**: mean number (±SEM) of scans when the foals performed different types of locomotor activity. **Unknown settings** (on the left): foals left alone in an unknown arena; **Known settings** (on the right): foals left alone in the same arena on the following day, the arena is now more familiar.

Both 2-year-old groups of horses expressed similar high levels of whinnies after social separation (unfamiliar settings: Mann–Whitney U test: *N*_early handled_ = 9; *N*_control_ = 8; *z* = −0.1, *p* = 0.89; familiar settings: Mann–Whitney U test: *N*_early handled_ = 9; *N*_control_ = 8; *z* = −1.36, *p* = 0.17; Figure 3A). However, in unfamiliar settings, the control group’s level of locomotor activity was significantly higher than that of the early handled group (Mann–Whitney U test: *N*_early handled_ = 9; *N*_control_ = 8; immobility: *z* = −0.24, *p* = 0.81; slow walk: *z* = −1.5, *p* = 0.14; active walk: *z* = 2.04, *p* = 0.03; Figure 3B). On the second day of test, when the settings were less unfamiliar, levels of locomotor activity of the two groups did not differ significantly even though control foals still tended to be more active (Mann–Whitney U test: *N*_early handled_ = 9; *N*_control_ = 8; immobility: *z* = 0, *p* = 0.96; slow walk: *z* = −0.34, *p* = 0.74; active walk: *z* = 1.74, *p* = 0.07; Figure 3B).

As stallions underwent castration when they were 1-year-old, we tested a potential effect of sex on the active locomotion. No difference could be evidenced in the level of active walk between males and females whatever the day of test (Mann–Whitney U test: *N*_females_ = 10; *N*_males_ = 7; unfamiliar settings: *z* = −0.34, *p* = 0.74; familiar settings: *z* = 0.98, *p* = 0.31). The castration did not seem to interfere in the way horses react to social isolation.

## Discussion

One single prolonged bout of handling just after birth clearly affected later adult expression of stress. Interestingly, in very stressful situations, such as social separation associated with a novel object or a novel environment, both experimental groups of horses produced the same quantities of whinnies, showing that they were similarly distressed by social separation. On the other hand, early handled foals differed from control (unhandled) foals as their occurrences of active forms of locomotion such as trot or gallop were less frequent. These behaviors reflect contact or escape seeking and therefore are considered as the expression of a distress (Wolff et al., 1997; Visser et al., 2001; Yeon, 2012).

This is an especially major effect as in the meanwhile both groups lived in the same conditions, in social groups with little contact with humans, apart from feeding. Similarly, rats that experienced prolonged repeated maternal separation (several hours per day for 2 weeks) reacted to stress (e.g., forced swim test) by an increased immobility rate (Lehmann and Feldon, 2000). Locomotor inhibition has been considered as depression-like behavior in the forced swim test, the Porsolt, or tail suspension tests (Porsolt et al., 1977; Steru et al., 1985). It is considered to be the expression of behavioral despair (Porsolt et al., 1978). Individual differences in expressing active/passive strategies when facing challenging situations are generally interpreted as variations in personality traits. Thus, in tonic immobility tests, some pigs struggle while others stay immobile but tensed, but all reacted vocally (Erhard et al., 1999). These authors interpreted these responses as indications that all pigs found the situation challenging and that they reacted differently according to their personality traits. In view of our study, one can wonder whether these traits could be the issue of differences in early experience. Emotional reactivity can be modulated, for instance, by maternal behavioral characteristics to which individuals have been exposed in their early life (Francis et al., 2003; Richard-Yris et al., 2005). So, even though our experiment did not allow evaluating the personality traits of the subjects, it suggests that a single experience, very close to birth, may be enough to influence later behavioral reactions to stress.

Indeed, to our knowledge, the present data for horses are the first to demonstrate the effects of postpartum handling experience not so much on adult stress reactivity *per se*as on the way reactions to stress are expressed. Horses’ reactions to motion-inducing situations include postural/vocal components as well as locomotor components (Wolff et al., 1997; Visser et al., 2001). Long-term effects of early handling obviously affect the locomotor component, but not the other expressions of stress reactivity. The fact that, in the social separation situation, both groups of horses produce similar quantities of whinnies indicates a high level of stress. Whinnies are typical contact calls, mostly produced during separation and aiming to restore contact (Waring, 2003; Lemasson et al., 2009; Pond et al., 2010). It is a typical expression of stress in social separation tests (LeScolan et al., 1997; Wolff et al., 1997; Moons et al., 2005). Interestingly, tranquilization by acepromazine maleate induces a decrease of calling but no changes in locomotor components in separation tests (McCall et al., 2006). These locomotor components are not particular to emotional reactions but reveal increasing arousal (Wolff et al., 1997). Learned helplessness typically decreases an individual’s attempts to escape a situation, as in previous experiences, movements were of no use (Maier and Seligman, 1976). Some authors suggest that active postpartum handling created a situation of learned helplessness (Simpson, 2002). Indeed, foals struggle for a while (“protest”) before becoming motionless (“despair”) during this phase when they are lying on the ground and physically separated from their mother while, at that stage, they are supposed to be up, and suckling (Waring, 2003; Henry et al., 2009). Reports suggest that the young individual must play an active part in bonding with its mother in order to develop a secure attachment (Hausberger et al., 2007). Our results could be interpreted as foals may have learned that movements (struggling with legs) were useless for restoring contact during the handling procedure. Thus, when facing again social separation and novelty, when sub-adult, they may “renounce” seeking actively contact or escape and therefore reduce their active locomotion. In particularly stressful contexts, only the vocal component was expressed, reflecting real stress, while the locomotor component, previously affected by unsuccessful attempts to escape, was inhibited.

These findings are of primary importance as they encourage further reflections on long-term effects of routine handlings of humans and animals.

## Materials and Methods

The experiment was conducted at the “Station expérimentale de Chamberet” (French National Stud Farms, France). Both procedure and testing were conducted in accordance with the French regulations governing the care and use of research animals and have been approved by the scientific committee of the French National Studs. The experiment was performed in accordance with the European Communities Council Directive of 24th November 1986 (86/609/EEC).

### Subjects and early treatment

Subjects were 17 French Saddlebred foals (10 females and 7 males) and their dams (*Equus caballus*) born at the “Station expérimentale de Chamberet” (France) where they stayed during the whole experiment. A few days before parturition, mares were stabled in a 4 m × 4 m foaling stall where delivery took place. Delivery was not assisted and newborn foals received minimal care.

Foals were allocated to one of two following treatments according to sire and date of birth:

(1) A control group (*n* = 8; 6 females, 2 males): no postpartum handling.
(2) An experimental group (*n* = 9; 4 females, 5 males) that was subjected to a handling procedure within 10 min after birth and before standing up and first suckling.

This procedure is a “routine” procedure used in breeding farms and inspired by the “imprint training” proposed by Miller in 1991 (cited in Henry et al., 2009). During the procedure, foals were restrained and maintained in a recumbent position, while the experimenter stroked them all over the body and exposed them to novel tactile stimuli (towel, plastic bag, water spray). Each stimulus was repeated until foals remained immobile during the procedure. The procedure lasted on average 72.1 ±  3.4 min and was always performed by the same handler (S.H.). After this procedure, mares and foals were left undisturbed.

Both experimental and control groups were then kept under the same management: in groups with mares until they were 7-months-old (weaning) and then in one outdoor same-age group except for the winter periods when they were housed in groups of 6–7 foals (handled and unhandled foals were mixed) in 10 × 50 m adjacent pens. Water and roughage were provided *ad libitum* while concentrates were given in winter. At 1 year of age, males were castrated under anesthesia. Contact with humans was limited to food provisioning for the whole experimental period (2 years).

### Experimental tests at later periods

The horses were presented experimental tests when they were one and 2 years old to evaluate their emotional reactions to two forms of stress (social isolation and neophobia). During test periods, they were housed in adjacent 4 m × 4 m stalls and turned out from noon to 4 p.m. The animals knew the stalls as they had been housed there with their dams when they were younger. All experimental tests were performed by the same experimenter (C.S.) blind to the horses’ early treatment.

One-year-old horses were confronted (1) to a novel object (bright colored inflated balloon) in their familiar stall placed there when the subject was not there. Latency to approach and their activities were recorded every 10 s for 5 min (see also Lansade et al., 2008). (2) They were also placed in social isolation, i.e., left alone in their stall for 1.5 min while the other horses were turned out. The individual’s behavior was recorded every 5 s.

Two-year-old horses were presented in a circular outdoor arena (diameter: 18 m) covered with sand and underwent the following tests that had previously proved to be appropriate (Wolff et al., 1997; Hausberger et al., 2004):

− An open-field test (Hall, 1934): a horse was released alone in the unfamiliar arena for 10 min. Instantaneous sampling behavior was performed every 10 s, while rare or brief behaviors (e.g., whinnies) were recorded each time they occurred;
− An “arena test”: a horse was released alone in the now more familiar arena for 10 min. The recording procedure was similar to that used in the previous test.

All test observations were recorded on a voice recorder and later analyzed. The behaviors recorded were the same as in earlier studies (Wolff et al., 1997). The coder has been trained by other members of the team and performed joint observations in a previous experiment (Henry et al., 2012).

### Statistical analyses

Analyses used non-parametric statistical tests (Siegel and Castellan, 1988), as normality of data was not ensured. Locomotor behaviors were divided into three categories: immobility (vigilance + observation), slow walk (exploration), active locomotion (active walk, defined as a sustained walk with the head above withers level looking ahead or around, trot, gallop, canter). Mann–Whitney U tests compared two independent samples (e.g., group differences). All tests were performed on discrete variables (number of occurrences, latencies). The threshold of significance was fixed at α = 0.05. *P*-values between 0.05 and 0.10 were called trends.

## Conflict of Interest Statement

The authors declare that the research was conducted in the absence of any commercial or financial relationships that could be construed as a potential conflict of interest.
